# Hemoglobin concentration and anemia diagnosis in venous and capillary blood: biological basis and policy implications

**DOI:** 10.1111/nyas.14139

**Published:** 2019-06-23

**Authors:** Lynnette M. Neufeld, Leila M. Larson, Anura Kurpad, Sheila Mburu, Reynaldo Martorell, Kenneth H. Brown

**Affiliations:** ^1^ Global Alliance for Improved Nutrition (GAIN) Geneva Switzerland; ^2^ Department of Medicine the University of Melbourne Melbourne Victoria Australia; ^3^ Saint John's Medical College Bangalore India; ^4^ Independent Consultant for GAIN London UK; ^5^ Rollins School of Public Health Emory University Atlanta Georgia; ^6^ Bill & Melinda Gates Foundation Seattle Washington

**Keywords:** hemoglobin, anemia, venous, capillary, diagnosis

## Abstract

Anemia is an important public health challenge and accurate prevalence estimates are needed for program planning and tracking progress. While venous blood assessed by automated hematology analyzers is considered gold standard, most population‐based surveys use point‐of‐care diagnostics and capillary blood to estimate population prevalence of anemia. Several factors influence hemoglobin (Hb) concentration, including human and analytic error, analysis method, and type of instrument, but it is unclear whether biological variability exists between venous and capillary blood. The objective of this paper was to systematically review sources of Hb variability and the potential biological basis for venous and capillary differences. We use data from a recent survey in the state of Uttar Pradesh, India, to illustrate the implications on anemia prevalence estimates. Significant differences in Hb concentration between capillary and venous blood samples are common. Most but not all find capillary Hb concentration to be higher than venous. Instrument/method variability and human error play an important role, but cannot fully explain these differences. A normative guide to data collection, analysis, and anemia diagnosis is needed to ensure consistent and appropriate interpretation. Further research is needed to fully understand the biological implications of venous and capillary Hb variability.

## Graphical abstract

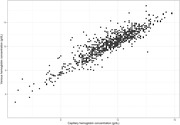

## Introduction

Anemia, defined as low blood hemoglobin (Hb) concentration, is a significant health problem globally and is associated with adverse health effects, and wide‐ranging social and economic development issues.^1^ According to the World Health Organization (WHO), 1.62 billion people worldwide are affected by anemia, with the highest prevalence observed in low‐ and middle‐income countries. Across Africa, it is estimated that 32% of children less than 5 years of age and 44% of pregnant women are anemic.^2^ In high‐income countries, anemia still affects 11% of children less than 5 years of age, 16% of women of reproductive age (WRA), and 22% of pregnant women.^3^

Anemia in pregnancy is associated with low birth weight and increased risk of maternal and perinatal mortality.^4^ Low birth weight is associated with malnutrition in children as well as increased later life risk of chronic diseases, such as hypertension and cardiovascular disease. Addressing prenatal anemia has been identified as a global health priority.^5^, ^6^ In children less than 5 years of age, iron deficiency anemia (IDA) adversely affects cognitive and motor development. In adult women, fatigue and decreased productivity have also been documented. In the long term, this can have a significant negative impact on educational performance, which can lead to underperformance or increased school dropout rates, impede workforce development, and potentially impact a country's economic productivity. The median annual economic loss due to IDA in 10 developing countries has been estimated to be $16.8 per capita or approximately 4% of GDP.^7^

In contexts, where the prevalence of anemia is high and there may be constraints to individual diagnosis and treatment (e.g., weak health systems or low health system utilization), public health measures are recommended.^8^ Accurate estimates of the magnitude and distribution of anemia in populations are essential to plan such efforts. Similarly, tracking progress toward targets for anemia reduction, such as the World Health Assembly target of reducing anemia by 50% in women by 2025,^9^ requires confidence in the quality and consistency of estimates from different sources/surveys over time and geographic regions.

The gold standard for Hb assessment is the cyanmethemoglobin (CMH) method.^10^ A number of automated hematology analyzers are now available and have been validated against this gold standard.^1^, ^16^ While any whole blood sample (i.e., venous or capillary) could be used, laboratory and clinic‐based methods usually rely on venous blood. Thus, venous blood assessed by automated hematology analyzers is considered the gold standard for anemia diagnosis. In population‐based surveys, however, resources (time, technical, and/or financial) may be a constraint to the use of such methods, as might the acceptability and feasibility of venous blood collection. Currently, the most common protocol for Hb assessment in field settings uses capillary blood obtained via finger‐prick, assessed on Hemocue^®^ (Hemocue AB, Angelholm, Sweden), a battery‐operated portable point‐of‐care photometer. There are several Hemocue models, including Hemocue B‐Hb, Hemocue Hb 201+, and Hemocue Hb 301.^11^ The Hemocue measures Hb using model‐specific microcuvettes.^12^ The Hemocue Hb 201+, for example, uses cuvettes, which contain a sodium deoxycholate reagent that leads to hemolysis of red cell membranes, releasing Hb from the red cells. The Hb iron is then converted by sodium nitrate from ferrous to ferric acid, to form a stable azidemetHb, which is detected at 570–880 nm.^11^ The Hb 301 does not contain these active ingredients and Hb concentration is measured by measuring the absorbance at an Hb/oxyHb isometric point.

Many published studies have compared Hb concentration and resulting anemia prevalence estimates using blood samples of different origin (venous and/or capillary), and different analysis methods and protocols. Table 1 provides an overview of comparisons from the literature and sources of variability that were assessed, assuming that error, both human and analytic (due to poor technical or quality control), has been minimized. Blood sample collections, particularly capillary samples, are highly subject to measurement error, if adequate attention is not given to quality criteria. Similarly, instruments, particularly those used in the field, such as Hemocue, require regular and careful maintenance and calibration, the lack of which can be an important source of error (see Table 3 for details of sample quality control criteria). Many of these sources of measurement error can be controlled/minimized in research and surveys with clear and detailed protocols and adequate training and quality control measures.

**Table 1:** Overview of comparisons in hemoglobin concentration and/or anemia prevalence from studies using samples of venous and/or capillary blood and different analysis methods and protocols

Type	Blood sample source	Reference method	Method tested/validated	Potential source(s) of variability assessed*a*	Ref
1	Split venous sample	Cyanmethemoglobin	Automated hematology	Analytic	–
2	Split venous sample	Cyanmethemoglobin or automated hematology	Hemocue	Analytic	22, 24, 25, 33, 37
3	Sequential capillary	Cyanmethemoglobin or automated hematology	Hemocue	Analytic Biological ○ Between drops	21
4	Sequential capillary	Both samples on any single method/model	–	Biological ○ Between drops	33
5	Venous (or capillary)	Hemocue one model	Hemocue different model	Analytic	–
6	Venous and capillary	Cyanmethemoglobin or automated hematology (venous)	Hemocue (capillary)	Analytic Biological ○ Venous versus capillary	19, 20, 23, 28, 29, 30, 31, 32, 48
7	Venous and capillary	Both samples on any single method/model	–	Biological ○ Venous versus capillary	–

*^a^*Assuming that human error has been minimized.

Studies have also compared point‐of‐use diagnostic tools, particularly Hemocue with gold standard laboratory methods, and in venous versus capillary blood. Results vary with some studies finding capillary Hb concentration higher than venous and others the contrary (Table 2). Unfortunately, the methodology used in most (11/20 studies, type 6 from Table 1) does not permit the distinction of instrument/method variation from biological variation (i.e., venous versus capillary). Several of these studies provide limited detail to permit an assessment of the extent to which human and analytic error was minimized.

**Table 2:** Overview of study results presenting a comparison of hemoglobin concentration in capillary and venous blood samples

	Number of studies by comparison type identified in Table 1			
	Type 6	Type 7 (Hemocue)	Type 7 (lab)	Total
Venous Hb not different from capillary Hb	0	1	2	3
Venous Hb > capillary Hb	2	1	1	4
Venous Hb < capillary Hb	11	4	5	20

Hb, hemoglobin concentration.

Such an error may at least in part explain the diversity of findings related to differences in venous and capillary blood shown in Table 2. Whether in addition there is a biological basis for a difference in venous and capillary blood is not yet well understood nor are the potential implications of any such difference for the accurate estimation of anemia prevalence. Such a difference, if it exists, could have important implications for the classification of severity of anemia and resulting prioritization/choice of strategies to address it and in the ability to track progress over time from surveys that might use diverse methodologies.

The objective of this review is to provide an overview of current knowledge related to the difference in Hb concentration assessed in venous and capillary blood and its implications for assessing the burden of anemia in populations. First, we illustrate the potential issues using data from a recent survey in Uttar Pradesh (UP), India, in which venous and capillary samples were collected on the same women. Following this, we present a systematic literature review of the topic (methods described below) and end with a discussion of the implications for anemia diagnosis in populations and identify several research gaps.

## Case study of venous and capillary Hb concentration from a cross‐sectional survey of nonpregnant WRA living in UP State, India

### Study setting, participants, design, and data collection

The study was led by the Global Alliance for Improved Nutrition in collaboration with the St. Johns Research Institute (SJRI), Bangalore, the Sanjay Gandhi Post Graduate Institute of Medical Sciences (SGPGI), Lucknow, Cornell University, and the India Nutrition Initiative. The study was authorized by the State Ministry of Health authorities and the proposal was reviewed and approved by the institutional review boards of SJRI and SGPGI. Data collection took place between October and December 2016.

We conducted a cross‐sectional survey of nonpregnant WRA 18–49 years of age living in UP as a baseline for the impact evaluation of a public‐sector nutrition program (subsidized sale of double fortified salt (DFS) through the state‐run fair price shops). The evaluation is registered with 3ie's Registry for International Development Impact Evaluations (RIDIE‐STUDY‐ID‐58f6eeb45c050). Five of the 10 districts prioritized for the DFS program were randomly selected to be part of the baseline (using simple random sampling (SRS) with a random number generator). Five matched control districts were then selected from among districts adjacent to each intervention district using SRS. Within each district, 20 villages or wards (a term used for urban neighborhoods in India) were randomly selected using SRS from among all villages and wards in the 2011 Census of India. Within each ward, one Census Enumeration Block (CEB) was randomly selected using a random number generator. Within each of village and each CEB, 5−8 WRA were selected to be included in the survey using a modified Random Walk Method for a total of 1300 WRA.^13^ Eligibility was based on the women's age (between 18 and 49 years of age), and her status as a mother of a child 6–59 months of age.

Written informed consent (or thumb print from participants unable to write their name) was obtained from all caregivers participating in the survey after receiving full information related to objectives, procedures, and risks. If the woman did not initially consent to any portion of the survey (household questionnaire, anthropometric measurements (not described here), or venous blood collection), the household was replaced; capillary blood sampling was also included, but the acceptance was not a condition for inclusion.

Blood collection was performed in each participant's home by six teams of three trained specialists, including a phlebotomist, an assistant, and a data recorder. Following the household survey, a 10‐mL venous sample was collected from each woman. Four milliliters of venous blood was collected in plastic spray‐coated K_2_EDTA tubes from BD Vacutainer^®^. The remaining blood was collected to assess the status of various micronutrients, the results of which will be reported elsewhere. After slowly inverting the vial twice, a syringe was used to collect a drop of whole blood from the tube and the drop was placed on a plastic slide. The phlebotomist then filled a microcuvette with the drop and immediately tested the Hb concentration using the Hemocue Hb 201+ analyzer (Hemocue AB). A capillary sample was then collected from the woman's fingertip using standard procedures,^14^ specifically by removing the first drop and filling the cuvette with a single blood drop. Hb concentration was measured using the same Hemocue as for the venous sample. Both samples, not necessarily from the same arm, were taken while the woman remained in sitting position within 15 min of each other. Ferritin was measured using sandwich electrochemiluminescence immunoassay (ECLIA) ELECSYS 2010 (Roche Diagnostics, Mannheim, Germany). CRP and AGP were measured using immunoturbidimetry: Cobas Integra 800 for CRP and Hitachi 902 for AGP (Roche Diagnostics).

Clear blood collection and handling guidelines were developed, and although all phlebotomists were experienced, we conducted an extensive training. Venous and capillary Hb measurements were performed on the same Hemocue immediately after collection, as described above. Each phlebotomist used the same Hemocue analyzer throughout the data collection period of this study (i.e., six phlebotomists and six Hemocues in total during the study). Every morning, three control fluids (Biorad: low, medium, and high) were used to assess the calibration of the Hemocue; no Hemocue required replacing due to out‐of‐range values during data collection.^12^ Every evening, the Hemocue was cleaned using standard procedures.^14^ The remaining blood samples were prepared and stored using standard procedures for laboratory analysis of multiple biomarkers of micronutrient status (not reported here). Monitoring of data collection and handling quality occurred through the study, using several criteria to assess data quality. There were no important deviations from the sample collection and handling protocol, as described in Table 3.

**Table 3:** Level of compliance to good practice techniques for blood collection and analysis in field settings

	Good practice	Level of compliance	Notes
Controls	Perform daily QC testing by measuring and recording the results for each of the low, normal, and high‐range control vials	+++	Performed and noted every morning prior to leaving for the data collection.
Equipment	Phlebotomists use powder‐free gloves	+++	Powder‐free gloves provided to phlebotomists.
	Storage of microcuvettes in humidity‐controlled container until ready to use	+++	Microcuvettes were kept in a sealed container, which was kept in a dark equipment bag. Microcuvette container typically used in less than a week. Data collection performed from September to December during temperate climate (10−25 °C).
	Clean Hemocue daily	++	Phlebotomists instructed to clean Hemocue every evening.
Fingerstick and Hemocue procedure for capillary sample	Use middle or ring finger	++	Phlebotomists trained to prick middle or ring finger, and finger with no ring.
	Massage finger	++	Phlebotomists trained to stimulate blood flow by massaging finger from knuckle to fingertip.
	Wipe finger with alcohol	++	Phlebotomists instructed to let alcohol air‐dry before sticking the finger.
	Stick the selected finger with the lancet	++	Phlebotomists instructed to prick the side of fingertip with enough pressure to get proper blood flow.
	Wipe away the first drop of blood with a clean gauze pad	++	
	Fill the microcuvette with the third drop	++	Phlebotomists instructed to fill the microcuvette with the second or third drop.
	Let blood drop flow into the microcuvette	++	Phlebotomists were trained not to “milk” the finger so as not to dilute the blood drop with interstitial fluid.
	Fill microcuvette fully	++	Phlebotomists were trained to fill microcuvette fully in one continuous process.
	Inspect cuvette for air bubbles	++	Phlebotomists were trained to avoid air bubbles.
Procedure for Hemocue assessment on venous sample	Mix venous blood in vial	++	Phlebotomists were trained to gently invert vial twice before taking a drop of blood.
	Using a pipette, place drop of blood on the plastic film	++	Phlebotomists were trained to place a drop of blood on plastic slide.
	Fill microcuvette in one continuous process with drop of blood	++	Phlebotomists were trained to fill microcuvette fully in one continuous process.
	Inspect cuvette for air bubbles	++	Phlebotomists were trained to avoid air bubbles.

note: Developed based on documented sources of error and good practice guides for sample collection and processing.^11^, ^17^, ^18^

+++, 100% compliance (observed); ++, phlebotomists trained on proper protocol, but daily observation of all not feasible. High compliance concluded based on daily observations of quality monitor.

For both the venous and capillary samples, anemia was defined as Hb concentration <12 g/dL, mild anemia as 11 g/dL ≤ Hb < 12 g/dL, moderate anemia as 8 g/dL ≤ Hb < 11 g/dL, and severe anemia as Hb < 8 g/dL. No adjustment for elevation was required because all elevation was below 1000 m. Iron deficiency was defined as ferritin <15 µg/L after the adjustment for CRP and AGP using the BRINDA regression approach.^15^, ^16^

The objective of the current analysis was to examine the relation between Hb concentrations from venous and capillary samples, therefore, only women with both measures were included in the analysis. Means, standard deviations, and prevalence statistics were derived using standard procedures on SAS^®^ 9.4 (SAS Institute, Cary, NC). Paired *t*‐tests were used to compare means across groups. Pearson's correlation coefficients and a Bland–Altman plot were developed to demonstrate the relation between capillary and venous sample Hb concentrations. Finally, an ANOVA was used to examine differences in mean venous and capillary Hb concentrations between phlebotomists.

## Results

**Figure 1:**
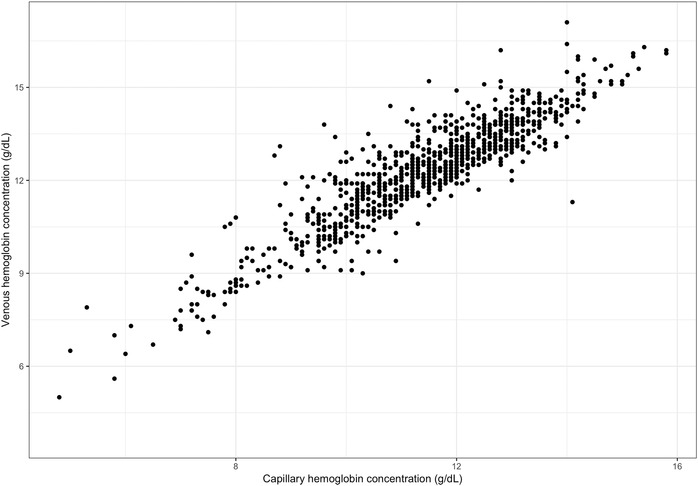
Correlation matrix of hemoglobin concentration between capillary and venous blood samples from 997 nonpregnant WRA from Uttar Pradesh, India.

**Figure 2:**
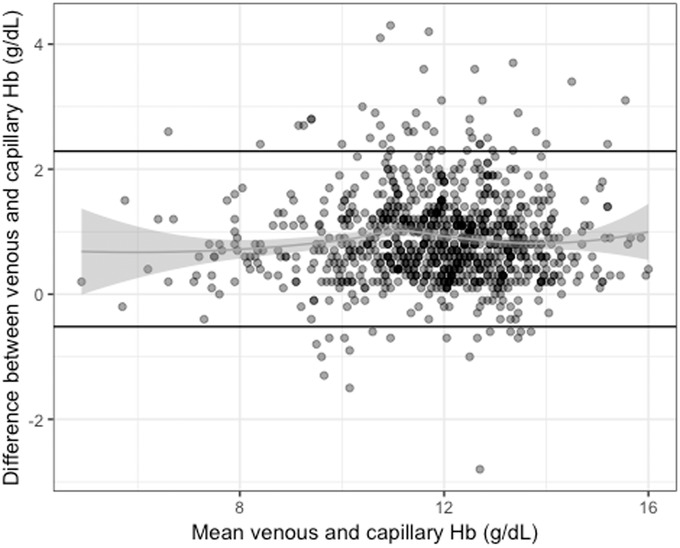
Bland−Altman plot for difference between venous and capillary hemoglobin concentration in 997 nonpregnant WRA. Dark horizontal lines represent 95% confidence intervals around the mean difference between venous and capillary hemoglobin.

Venous and capillary Hb concentrations were available for a total of 997 nonpregnant women. The correlation between venous and capillary Hb concentrations was high (*r =* 0.91, *P <* 0.001) (Fig. 1). Mean Hb concentrations were significantly higher in venous than capillary samples for the full sample (mean ± SD: 12.3 ± 1.7 versus 11.4 ± 1.6 g/dL, *P <* 0.001), and within each category of anemia, using the venous sample to define anemia (Table 4). A similar pattern was seen among iron deficient and iron sufficient women compared with the full sample (results not shown). The Bland−Altman plot for differences between venous and capillary Hb concentrations is presented in Figure 2 and indicates heteroskedasticity; the magnitude of the difference between venous and capillary samples is not consistent across the range of Hb concentrations—with the difference being greater at higher venous Hb concentrations.

**Table 4:** Hemoglobin concentration in capillary and venous blood samples in 997 nonpregnant women of reproductive age*a*

	Venous (g/dL)	Capillary (g/dL)	Paired *t*‐test
Full sample	12.3 ± 1.7	11.4 ± 1.6	<0.001
No anemia	13.3 ± 0.9	12.3 ± 1.1	<0.001
Mild anemia	11.5 ± 0.3	10.7 ± 0.6	<0.001
Moderate anemia	9.9 ± 0.8	9.3 ± 1.0	<0.001
Severe anemia	7.1 ± 0.8	6.5 ± 0.9	0.003

note: Anemia categories defined using the venous sample.

*^a^*Values are mean ± SD.

Using venous samples, anemia prevalence was 35.2%; using capillary samples, anemia was 59.2% (Table 5). The majority of misclassified individuals were diagnosed as anemic using capillary, but nonanemic using venous (24.4%). Only four women were diagnosed as nonanemic using capillary, but anemic using venous blood. When anemia was categorized into mild, moderate, and severe, the majority of misclassification resulted from classifying nonanemic women (using venous blood) as mildly anemic (using capillary) (20.0%) and classifying mildly anemic women as moderately anemic (12.4%). Approximately 52% of women were iron deficient; the pattern of classification as anemic and not in venous versus capillary samples did not vary among women with and without iron deficiency (data not shown). To explore whether the results presented here varied by a phlebotomist, we assessed mean Hb concentration and mean difference between venous and capillary Hb by the phlebotomist. No significant differences in mean or standard deviation of venous Hb concentrations were observed among the six phlebotomists; however, significant differences emerged when comparing mean capillary Hb concentrations among phlebotomists (*P* = 0.03). A box plot of the difference between venous and capillary samples from each phlebotomist (Fig. S1, online only) illustrates that particularly two (phlebotomists 2 and 6) had inconsistent results between venous and capillary samples.

**Table 5:** Anemia prevalence using venous and capillary blood samples in 997 nonpregnant women of reproductive age*a*

			Venous sample			
			Any anemia			
			No	Yes	Total	
Capillary sample	Any anemia	No	40.4 (403)	0.4 (4)	40.8 (407)	
		Yes	24.4 (243)	34.8 (347)	59.2 (590)	
		Total	64.8 (646)	35.2 (351)		
		Venous sample				
	Severity	No anemia	Mild anemia	Moderate anemia	Severe anemia	Total
Capillary sample	No anemia	40.4 (403)	0.4 (4)	0	0	40.8 (407)
	Mild anemia	20.0 (199)	5.0 (50)	0.1 (1)	0	25.1 (250)
	Moderate anemia	4.4 (44)	12.4 (124)	13.6 (136)	0	30.5 (304)
	Severe anemia	0	0	1.8 (18)	1.8 (18)	3.6 (36)
	Total	64.8 (646)	17.8 (178)	15.5 (155)	1.8 (18)	

*^a^*Values are % (N).

### Discussion and conclusions

Data from this cross‐sectional survey of nonpregnant WRA living in UP show significant differences between Hb concentrations measured with a Hemocue using venous compared with capillary blood samples. Hb concentrations from venous samples were consistently higher than from capillary samples resulting in a mean difference of 0.9 g/dL (SD = 0.7). Prevalence of anemia was significantly higher using capillary samples than venous samples (59.2% versus 35.2%), with no variability in this relationship among iron deficient and iron sufficient women. The magnitude of this difference has potentially important consequences for conclusions related to the importance of anemia as a public health problem in the state. The sizeable differences in anemia diagnosis between the two methods may at least in part be because the mean Hb concentration in the population is close to the cutoff level for anemia (i.e., the cutoff for diagnosis of anemia is 12 g/dL and the mean venous Hb is 12.3 g/dL). Thus, a small difference between the Hb concentrations of the two types of samples can result in a different anemia classification. Specifically, according to the WHO classifications, based on estimates from venous samples, UP would be classified as having a moderate public health problem of anemia, while the use of the capillary estimate would lead to a conclusion that anemia is a severe public health problem.

The observed training and ongoing field methods monitoring results permit a reasonable conclusion that field procedures for the collection and processing of venous and capillary blood samples were relatively well followed. That said, the diversity in mean Hb concentration difference (venous–capillary) by the phlebotomist and the significant difference in mean Hb from capillary samples suggests that the capillary samples are likely more prone to measurement error. Furthermore, because differences between phlebotomists were observed with capillary Hb but not venous Hb concentrations and phlebotomists used the same Hemocue throughout data collection, we conclude that this error is more likely human rather than analytic (i.e., the phlebotomist and not the Hemocue itself). Whether this error can account for the entire difference between venous and capillary samples documented in this study cannot be assessed from the information available here. It is for this reason, and to specifically address the question of whether there is a biological basis for a difference between Hb concentration in capillary and venous blood that we now turn to a review of the literature.

## Sources of variation in Hb concentration between venous and capillary blood samples: literature review

This literature review sought to explore variation in Hb concentration measured in capillary blood versus venous blood, and the possible explanations for this observed variation. We sought to isolate the origin of that variation, separating that which may be due to human error (e.g., poor sample collection and processing technique) or instrument error (e.g., analytic failure and lack of calibration), from that which is due to instrument/method variability (i.e., differences between analysis technique that cannot be attributed to human error), and biological variability, focusing specifically on variation between venous and capillary samples that cannot be attributed to human or analytic error. Below, we articulate the key questions addressed in this review, using as a basis the types of comparisons and error as described in Table 1. Note that not all comparisons are explored in detail. The first, type 1 (assessment of analytic error between a CMH as reference methods and automated hematology on a split venous sample), is assumed as proven and literature was not reviewed here. Others, for example, type 3 (drop‐to‐drop variability and/or variability between instruments from sequential capillary samples), is not critical for our primary objective (i.e., venous versus capillary comparisons). The latter was considered however, if appropriate as additional possible explanations for the results of some comparisons.

1. To what extent do Hb concentration and anemia diagnosis differ as a result of instrument/method variability, that is, between automated hematology analyzers or laboratory methods and Hemocue (comparison type 2 from Table 1) or between different models of Hemocue devices (comparison type 5 in Table 1)?
2. What is the variability in Hb or anemia diagnosis in venous versus capillary blood?
3. Is there a biological basis for a difference in Hb or anemia diagnosis in capillary versus venous blood (comparison type 7 in Table 1)?

### Search criteria and approach

A broad search strategy was developed to answer the questions listed above, using the terms “hemoglobin,” “anemia,” “laboratory,” “field,” “diagno^*^,” “measure^*^,” “method” and “hemocue OR portable OR photometer,” “capillar^*^,” and “venous OR vein.” The search strategy included variations in the spelling of hemoglobin and anemia and different descriptions of hemoglobin measurement or assessment of anemia status. In addition, terms, such as capillary, which may appear in papers as “capillaries” or “capillary,” were truncated to ensure these differences were captured in the papers returned from the search. NCBI PubMed was the primary search engine used. Criteria were then refined to narrow the scope and further studies were included through cross‐referencing.

**Figure 3:**
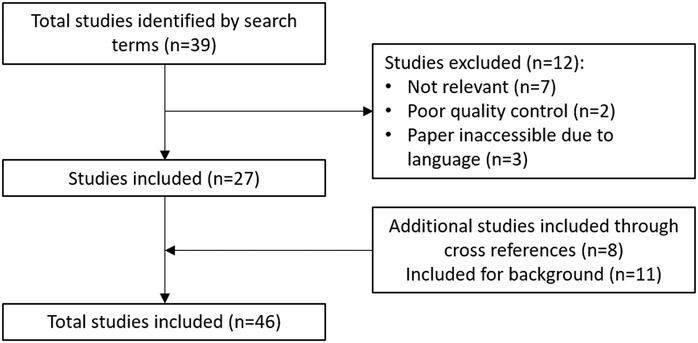
Flowchart of the systematic literature review process.

In an effort to include only those studies for which we were confident that human and analytic error had been minimized, the quality of each study was assessed using the same criteria outlined in Table 3. These criteria were compiled from documented sources of error in field blood collection and processing methodologies and published good practice guidance for the same.^11^, ^17^, ^18^ Using these criteria, we made an assessment of whether the studies met the majority of the quality control criteria (and thus labeled as acceptable) and those that showed poor quality control or failed to mention quality assurance measures and thus could not be adequately assessed. The latter group was marked as unacceptable and not included in the literature review. A search of PubMed yielded close to 9000 results. Thirty‐nine abstracts were identified for further review of the full paper; of these, 27 were included in this systematic review. Eight additional studies were identified through references of the included papers and 11 others provided information relevant to the review (but not necessarily Hb comparisons) (Fig. 3). A number of studies provided multiple comparisons and are thus included to address more than one of our research questions.

Given that laboratory methodologies are well established, we did not review the literature that compares the CMH method with automated hematology analyzers (comparison type 1 in Table 1). The majority of the studies reviewed that included laboratory assessment used automated hematology analyzers, while some older studies used the CMH method—both are included in our review. For field‐based methods, we found only one study that met the inclusion criteria that used a device other than the Hemocue. This study, using the STAT‐SiteM^Hgb^ and the URIT–12, done in a community clinic field setting in Durban, South Africa, found that both devices showed low accuracy when compared with a laboratory reference standard;^19^ we, therefore, excluded it from this review.

#### To what extent do Hb concentration and anemia diagnosis differ as a result of instrument/method variability, that is, between automated hematology analyzers or laboratory methods and Hemocue (comparison type 2 from Table 1), or between different models of Hemocue machine (comparison type 5 in Table 1)?

As noted above, the laboratory methods CMH and/or automated hematology analyzers have been established as the gold standard for Hb assessment, and field methodologies, using the Hemocue, have been assessed in comparison to them. Studies undertaken under controlled laboratory conditions using split venous samples have shown that the Hemocue has high correlation (0.96 with relative error of <3.5%) with Hb measured using the gold standard direct CMH method.^18^, ^19^, ^20^, ^35^ Studies report high specificity for the diagnosis of anemia in field methods using laboratory methods as the gold standard (94.2%)^10^, ^22^ but lower sensitivity ranging from 56% to 95%,^30^ implying that some individuals with anemia are not correctly diagnosed as such using Hemocue.

After eliminating those that did not pass our minimum quality review, we identified additional studies that assessed Hb measured by Hemocue and a comparison method under field conditions in both school‐aged children and adults, all of which reported correlations lower than those reported in laboratory settings.^21^, ^22^, ^23^ Correlations with standardized methods under field conditions ranged from 0.61 to 0.95. Unfortunately, some studies reported only these correlations and not the actual differences in Hb concentration. Three studies reported higher mean Hb concentration using field methods than laboratory methods.

Some studies have found that interoperator error can influence the reliability of Hb measured using the Hemocue device. A cross‐sectional survey measuring Hb in rural Cambodian women found significant variability in the measurement of Hb among device operators using Hemocue Hb 301, despite high‐quality training.^26^ Unlike the study reported above in UP, not all operators used a single Hemocue device over the course of the study, and, therefore, the difference observed may have been due to the Hemocue itself, the operator or both. As per the authors’ assessment, other sources of variability and error in Hb, for example, due to humidity, storage of microcuvettes, or transport of the Hemocue, could not be ruled out in this study.

In order to delineate the variability conferred by Hemocue device, several studies have compared the measurement of Hb in capillary or venous blood across similar measurement devices. A study in indigenous Mexican children, which compared Hb measurement of Hemocue B‐Hb with Hemocue Hb 201+, showed 0.995 device correlation in capillary blood, and 0.996 correlation in venous blood, suggesting that Hemocue model is unlikely to account for the variability between capillary and venous blood Hb.^23^ In contrast, a study comparing the Hb in capillary blood using the Hemocue Hb 201+ and Hemocue Hb 301 models found that measurement of Hb in capillary blood with Hemocue Hb 301 produced Hb measurements 3.3% higher than the Hb 201+ model indicating that the choice of Hemocue model may indeed influence Hb concentration.^11^ A third study compared Hb measured in venous blood using a laboratory hematology analyzer and capillary blood on two models of the Hemocue. Mean venous Hb (12.8 g/dL) was lower on average than any of the capillary assessments on either Hemocue (Hemocue B‐Hb: 13.5 g/dL; Hemocue Hb 201+: 13.7 g/dL). They also assessed the venous blood on the two Hemocue devices, still finding higher Hb than venous samples on the laboratory device, albeit less so (Hemocue B‐Hb: 13.0 g/dL; Hemocue Hb 201+: 13.2 g/dL). The authors conclude that while the Hemocue devices may confer a very small degree of measurement error in Hb compared with the gold standard, the device itself does not fully explain the higher Hb in capillary compared with venous samples.^11^

Thus, in answer to our first question, we conclude, based on a limited number of studies, that Hemocue can provide an accurate assessment of Hb concentration compared with laboratory methodologies, that is, the instrument/method variability is minimal when split venous blood samples are tested.

#### What is the variability in Hb or anemia diagnosis in venous versus capillary blood?

The majority of studies that have been published comparing Hemocue and laboratory methods or venous and capillary blood have used a mixed protocol, that is, assessing venous blood using laboratory methods and capillary blood using portable photometer. As noted in Table 1, even if limited to those studies that documented good sampling procedures, this does not permit the distinction of variability due to analytic differences in method from variability in venous and capillary blood. Although the high correlation between Hemocue and laboratory methods noted above from studies using split venous samples provides some confidence that instrument/method variability can be minimized, one cannot fully attribute differences between venous and capillary samples to biological variability.

**Figure 4:**
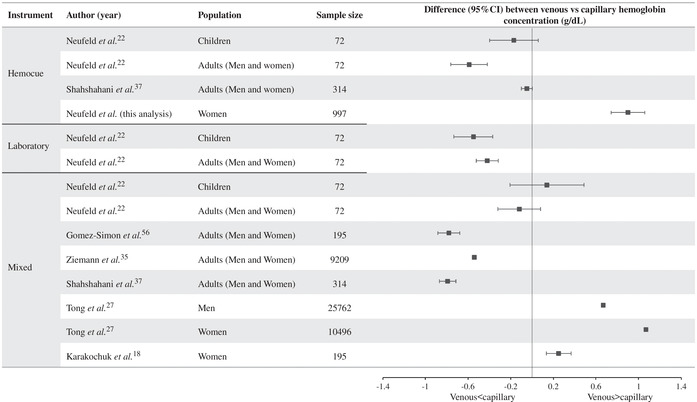
Forest plot of the difference in hemoglobin concentration in venous and capillary blood by analysis method (both by Hemocue, both in laboratory, or mixed) and population group. Estimates are difference (95% CI) in venous minus capillary hemoglobin concentrations. 95% CIs for Ziemann *et al*.^35^ in adults are (−0.549 to −0.531); and for Tong *et al*.^27^ in men and women are (1.062–1.078) and (0.658–0.682), respectively.

The results of these studies that have used “mixed” analysis methods vary, some finding higher Hb in capillary blood assessed by Hemocue,^22^, ^27^, ^28^, ^29^, ^30^, ^31^ while others found higher Hb in venous blood measured using laboratory methods.^32^, ^33^, ^34^ A large study in 1014 male and female Italian blood donors found that Hb measurement in capillary blood using Hemocue was higher than Hb measured in venous blood using laboratory analyzers.^32^ Similarly, a large study in Germany involving 9209 blood donors found that although measurements of capillary and venous blood Hb were correlated (*r =* 0.87, *P* ≤ 0.0001), capillary blood conferred a higher estimate of Hb when compared with venous blood.^18^ In the vast majority of donors, the measures of Hb in the two blood sources differed by 1 g/dL or less and authors indicated that such a small difference was unlikely to be of clinical significance.^35^ In contrast, a large study in 36,258 paired samples of capillary and venous blood from Irish blood donors measured higher Hb in venous blood compared with capillary blood.^27^ The differences between venous and capillary samples for those studies that provided appropriate details are presented as a forest plot in Figure 4 **;** the lower panel shows results from the studies mentioned that used mixed results.

Whether there is a difference between Hb concentration in venous and capillary blood can be better examined in those studies that have analyzed samples from each site using a single analysis methodology, that is, both venous and capillary in the laboratory, or both on Hemocue. These results are shown in the upper two panels of Figure 4. One laboratory‐based study in 72 Mexican children and adults found that when adequate capillary sample collection technique was used, Hb in capillary blood was significantly higher than in venous blood, in both adults and children.^22^ On average, Hb estimated using Hemocue in capillary blood was 0.5 g/dL higher than in venous blood also analyzed on Hemocue, but considerably higher among adults (0.59 g/dL) than children (0.17 g/dL). However, in a small proportion of the sample (19% of adults and 21% of children), Hb was lower in capillary blood than venous blood. One study in Chinese adults found no difference in Hb between capillary and venous blood.^36^

Several other studies support the conclusion that on average, Hb concentration is higher in capillary than venous blood. One study undertaken in 33 paired venous and capillary samples also found a nonstatistically significant 1 g/dL higher Hb measured in capillary than venous blood when both samples were measured using Hemocue.^35^ Shahshahani *et al*.^37^ also found that paired venous and capillary blood samples measured by Hemocue showed slightly higher, yet no significantly different Hb measured in capillary than venous blood. In a study of hospitalized children aged 3 months–14 years, measurement of Hb in capillary and venous blood using a laboratory hematology analyzer found that capillary Hb values exceeded venous values by 2%.^38^ Similarly, a study undertaken in 1600 term and preterm neonates found that Hb measured in capillary blood using a standardized hematology analyzer was higher than Hb measured in venous blood using the same method^39^ in both term and preterm infants. Another study in a smaller number of neonates (*n* = 16) also found that Hb measured from capillary blood was 3% higher than that in venous blood.^38^ In a study of 29 pregnant women, researchers found that Hb measured in capillary blood using Hemocue was higher than Hb in venous blood measured by both Hemocue and CELL‐DYN 3000 (Abbott, Abbott Park, IL) hematology analyzers.^40^ Similar results have been reported for pregnant women from a field study in Jamaica.^24^

In response to our second question, we conclude that based on the limited number of studies available, it is likely that differences in venous and capillary samples cannot be attributed to instrument/method variability or human error alone. While there is some variability in the direction of that difference across studies, the majority find capillary Hb concentration to be higher than venous Hb concentration.

#### Is there a biological basis for a difference in Hb or anemia diagnosis in capillary versus venous blood?

The database on biological variation has found that there is bias associated with Hb measurement driven by biological variation.^41^ The consistency in the difference between Hb in capillary and venous samples across most studies identified (Table 1), even when other sources of variability have been controlled for to the extent possible, is certainly consistent with that conclusion. The lack of consistency, however, in the direction of that relationship begs the question of (1) the origin of the difference between venous and capillary samples, and (2) the factors that might influence this.

A study published in 1929 identified the Fahreus effect, which described a fall in the blood hematocrit flowing through narrow tubes below 300 µm in diameter.^42^ The authors conclude that blood flowing through the microvascular system (capillaries, arterioles, and venules) has reduced hematocrit and red cell count compared with larger blood vessels, such as the veins or arteries.^42^ While consistent with our results from UP and one large study,^27^ this shows the opposite trend of results across the majority of studies identified.

There are a number of factors that might modulate the difference between capillary and venous Hb, including Hb concentration (as noted above). The study by Neufeld *et al*. suggests that age may be a factor in the magnitude (greater difference among adults than children), albeit not the direction of that relationship. Differences may also exist by sex. Based on studies by Murphy, the female microvasculature system appears to contain more red cells, hematocrit, and Hb concentration than males,^43^ implying that capillary samples may be more consistently higher than venous among women than men. This modulation is suggested to be controlled by the sex hormones androgen and estrogen in males and females, respectively. Androgens are agents of vasoconstriction, whereas estrogen facilitates vasodilation.^43^ As such, it is this sex hormone−mediated difference in vasoconstriction or vasodilation of the microvascular system that results in the gender differences observed in the differences in Hb measured in venous and capillary blood. Vasodilation of the microvascular system allows passage of larger amounts of hematocrit and increases red cell count. Supporting this hypothesis, the difference between Hb measured in capillary and venous blood has been found to be higher in males than females, likely mediated by males higher overall body red blood cell count, contrasted with the reduced hematocrit, red cell count, and Hb in their microvasculature system, including their capillaries.^27^, ^43^ In the study of over 36,000 paired samples from Irish blood donors, the mean difference between Hb in capillary and venous blood differed, with a difference of 1.07 g/dL measured in males and 0.67 g/dL in females,^27^ again indicating a biological difference in male and female microvascular system, and subsequently, measurements of Hb in venous and capillary blood. Furthermore, the difference between capillary and venous Hb measurements in men and women is mediated by age, with the difference decreasing but staying relatively constant in men with increasing age, but increasing steeply after the age of 45 in women.^43^

Moreover, a study undertaken in women in Honduras and Bangladesh provides further support for a biological difference in Hb measurement blood source. This study identified significant intraindividual variability in Hb measured in capillary blood taken from the left and right hand (CV = 6.3%), and Hb measured blood over 4 consecutive days (CV = 7.0%). Additionally, the Hemocue was found to have high accuracy when compared with the laboratory gold standard, indicating that instrument error could be ruled out as a cause of the variability observed in the capillary measurements. As such, authors postulated that it was biologically plausible that capillary blood measurement would be more variable in Hb concentration across different sites and on different days due to variation in extracellular fluid across different sites or on different days. Although technicians were well trained, the authors indicated that more research would need to be undertaken to explore whether the 6−7% coefficient of variation observed is biologically normal, or whether further standardization of protocols could reduce this variability. Replicate capillary sampling has been suggested as a way of controlling for this variability, but the practicality and acceptability of doing so in a field setting has been questioned.

We conclude that there is substantial evidence to support a conclusion of biological variability between capillary and venous blood after accounting for device and sampling variability and/or error, the magnitude of which is likely variable by sex, possibly age, and other factors. Whether these additional factors can fully account for the lack of consistency in the direction of that relationship in a few studies is uncertain.

### Other potential sources of variability in Hb concentration

In addition to the measurement device, blood sample collection site, and methodological errors, there are a number of factors that have been reported to influence Hb concentration that, unless taken into consideration, might influence conclusions related to the venous versus capillary comparisons. For example, postural differences have also been found to influence Hb readings, with the measurement at standing positions being identified as resulting in the highest Hb reading, sitting giving rise to slightly lower Hb, and measurements in participants lying supine identified as the lowest.^27^ Standing is thought to cause fluids to shift into interstitial space causing hemoconcentration, while lying supine does the opposite leading to hemodilution. The supine position is associated with 5−10% lower Hb due to shifts in plasma volume from the extra to the intravascular space.^44^ Although blood in the field is often taken with patients seated, if the patients have been standing a long time prior to measurement of Hb, or have walked a long distance, the measurement of Hb could potentially be affected. Studies have also shown that Hb concentration is higher when blood was collected following a shorter seating time.^44^ However, these studies have focused on venous samples, and it would perhaps be of interest to explore whether this same pattern is observed in capillary blood.^41^

An association has been observed between humidity and the accuracy of Hemocue measurements,^11^, ^45^ most of which can be mitigated through careful procedures. The Hemocue Hb 201+ is especially affected by humidity. The devices’ instructions indicate that microcuvettes should be stored at temperatures of 15−30 °C and at 10−40 °C for the Hemocue Hb 301+. Storage of Hemocue Hb 201+ and Hemocue Hb 301+ microcuvettes in a closed box above temperatures 37 °C and 72% for up to 3 weeks did not lead to significantly higher Hb results.^11^ However, when Hemocue Hb 201+ microcuvettes were stored in an open box, rapid degradation of the reagent stored within microcuvettes was observed after 10 min, and the Hb results observed were significantly lower. However, this appears to be limited to Hemocue Hb 201+ and has not been observed in Hemocue Hb 301. Other studies have shown that Hb measurements taken using microcuvettes from a packet opened more than 12 days prior to measurement led to an overestimation of Hb when compared with new cuvettes.^10^ Furthermore, the difference in anemia prevalence as tested by new and old microcuvettes (13.2% versus 16.5%, respectively) was significantly different. The authors suggested that manufacturer's recommendation that microcuvettes can be used up to 2 months of breaking the seal does not seem to apply to humid climates. Additionally, studies have found seasonal variability in the difference between Hb measured in venous and capillary blood, with the difference of Hb measured in capillary versus venous blood increasing in winter and decreasing in summer.^27^

Intraindividual variability among subsequent blood drops in the capillary measurement of Hb has also been documented.^46^ As such, some studies have indicated that measurements of Hb in a single drop of capillary blood are not reliable.^46^, ^47^ In a study of 11 blood donors, where up to six drops of blood were taken from one fingerprick, Hb differed between drops by more than 1.6 g/dL. In comparison, a study that analyzed 40 paired samples of blood found that Hb measured in the first and fourth drops did not differ meaningfully (0.03 g/dL difference).^46^, ^47^ However, the mean difference between duplicate samples (one from each hand) was significant (4.7 g/L for the first drop and 5.7 g/L for the second drop). The study also found that pooled blood drops provided a more precise estimate when compared with venous samples obtained under ideal conditions from experiences of phlebotomists and suggested that pooling of capillary blood drops may provide a more accurate Hb measurement than a single drop. However, a study by Whitehead *et al*. showed that second and third blood drops provided more precise estimates of Hb in capillary blood and were comparable to pooled capillary blood Hb, supporting the Hemocue protocol that stipulates the importance of wiping away the drops of blood and measuring the third.^11^

Enhancing standardized procedures and ensuring the utilization in research and other surveys, including, for example, standardization of patient posture during venous blood sampling, or the recommendation given that patients should sit an approximate amount of time prior to measurement, among others could limit factors that affect greater comparability across assessments.

## Discussion and conclusions: implications for policymaking and further research

Based on this literature review, we conclude that there are many sources of variation in Hb estimation, many but not all of which can be minimized through high‐quality data collection and processing protocols and strong quality control procedures in research and surveys. Even when measurement and instrument error and variability has been controlled, the estimated mean in Hb concentration estimated from capillary samples will vary from those estimated from venous samples. This difference is likely related to true biological variability, the magnitude of which is likely to vary by age and sex of participants. However, our understanding of the biological causes of the variability in venous and capillary Hb is still minimal. Where quality issues have not been minimized, these differences are likely to affect conclusions related to the severity of the problem of anemia in countries.

Pooling drops of blood from a single capillary blood draw is an important component of minimizing variability due to blood sampling technique and is recommended as part of a nutrition field survey manual.^48^ Yet, many of the studies and surveys reviewed reported using single capillary blood drops for analysis or did not include sufficient details in methodology to determine whether single or multiple drops were assessed. Similarly, the effect of sample handling on different analysis methods, for example, length of time between collection and processing, has been inadequately assessed. Rigorous testing to standardize these methodologies and document the ability for capillary samples to approximate venous Hb concentration should be highest research priority. Reporting standards for studies and surveys reporting Hb concentration should also be established to facilitate data interpretation.

With the current interest in anemia and targets to track progress toward anemia reduction, there are a number of potential practical implications of these findings. First, the extent to which differences in the estimate of Hb concentration due to methodological differences across surveys/studies affects anemia prevalence estimates will depend on the mean and distribution of true Hb concentration. In populations, where mean Hb is lower and close to the cutoff to diagnose mild anemia, or there is greater variability in Hb concentration, the implications of measurement error and/or diversity in estimates due to diverse sampling and analysis protocols across surveys will be greater. This issue, therefore, is particularly important in exactly those populations for whom we seek to strengthen interventions and track progress. This difference is strikingly illustrated in the results from the UP case study presented here.

Second, beyond the estimations of prevalence, the difference may have important implications for tracking the progress of actions to reduce anemia prevalence. If such progress is tracked through impact evaluations or research studies, close attention can be paid to assure the comparability of results. This should include avoiding comparisons of Hb and anemia estimates made on field and laboratory studies and on venous and capillary samples. Where multiple data sources are compiled to track progress and diverse methodologies have been used to generate those, the issues could be very complex. Depending on the sampling and analysis protocol, the magnitude of difference between Hb estimates from venous and capillary samples, for example, may be within the range of what we expect in the impact from a number of nutrition interventions. In Table 6, we present an illustrative summary of the magnitude of impact that has been reported on Hb concentration from select studies, reviews, and meta‐analyses. This is not intended as a comprehensive review but rather to illustrate that the magnitude of the impact of many of the interventions implemented to reduce anemia in populations may have an impact within the variability that has been demonstrated between venous and capillary, and between analysis methods for blood samples. The point of this table is to highlight the critical importance of ensuring consistency in sampling and analysis methods, and ensuring that human error is minimized when seeking to track progress toward anemia reduction.

**Table 6:** Illustrative examples of the magnitude of impact on hemoglobin concentration from reviews of select interventions commonly implemented to reduce anemia prevalence in populations

Population	Nutrition intervention type	Mean difference (95% CI) in hemoglobin (g/dL)	Ref
Women, men, and children	Double fortified salt (iron, iodine)	0.374 (0.142−0.606)	49
Adolescents	Weekly IFA	0.224 (0.036−0.412)	50
	Daily IFA	1.007 (0.405−1.610)	
Children (6−23 months of age)	Daily iron supplementation	0.407 (0.282−0.533)	51
Nonpregnant women of reproductive age	Daily oral iron supplementation	0.530 (0.414−0.645)	52
Preschool and school‐age children	Iron‐containing multiple micronutrient powders	0.337 (0.094−0.580)	53
Women, men, and children	Food fortified with iron	0.42 (0.28−0.56)	54
Children	Iron supplementation	0.74 (0.61−0.87)	55

CI, confidence interval.

Finally, even where the error is minimized, the choice of sampling and analysis methodologies may have important implications for anemia prevalence estimates. We strongly recommend that a normative guidance document on Hb concentration assessment and anemia diagnosis be developed. This would set standards for sample collection and analysis and could provide suggestions for reporting standards to avoid comparison of results from a different sample where not appropriate. A brief on reading and interpreting results from varying sources could also be prepared for policymakers and nonexpert users of the information in countries. This would ensure that they are aware of these issues and make informed choices on the trade‐offs of collecting and comparing data from multiple sources.

A comprehensive research agenda should be developed and implemented as soon as possible. Some of the issues that should be addressed with high priority include:

1. The extent to which multiple drop capillary sampling techniques, and processing, such as time from collection to analysis, can fully mitigate the issues raised in this paper.
2. A review and or additional research to understand the clinical implications of the difference in Hb concentration between venous and capillary blood in otherwise healthy individuals.
3. Review of the implications of these differences for tracking progress to WHA goals in diverse country settings, where multiple data sources are relied on.

## Author contributions

L.M.N. and L.M.L. conceived the study, wrote the manuscript, L.M.N. holds the final responsibility for its content. L.M.L. led the meta‐analysis with the support of S.M. for the systematic literature review. A.K., R.M., and K.H.B. provided suggestions for the scope and approach of the review. All authors have read and approve the final version.

## Statement

This manuscript was presented at the World Health Organization (WHO) technical consultation “Use and Interpretation of Haemoglobin Concentrations for Assessing Anaemia Status in Individuals and Populations,” held in Geneva, Switzerland on November 29−30 and December 1, 2017. This paper is being published individually but will be consolidated with other manuscripts as a special issue of *Annals of the New York Academy of Sciences*, the coordinators of which were Drs. Maria Nieves Garcia‐Casal and Sant‐Rayn Pasricha. The special issue is the responsibility of the editorial staff of *Annals of the New York Academy of Sciences*, who delegated to the coordinators preliminary supervision of both technical conformity to the publishing requirements of *Annals of the New York Academy of Sciences* and general oversight of the scientific merit of each article. The workshop was supported by WHO, the Centers for Disease Control and Prevention (CDC); the United States Agency for International Development (USAID); and the Bill & Melinda Gates Foundation. The authors alone are responsible for the views expressed in this paper; they do not necessarily represent the views, decisions, or policies of the WHO. The opinions expressed in this publication are those of the authors and are not attributable to the sponsors, publisher, or editorial staff of *Annals of the New York Academy of Sciences*.

## Competing interests

The authors declare no competing interests. No funding was received by the authors for this work, except for S.M. who worked on a short‐term consultant contract with GAIN.

## Supporting information


**Figure S1**. Box plot of mean differences between venous and capillary samples by a phlebotomist. Horizontal box lines represent median and interquartile ranges. Diamonds represent means hemoglobin concentrations for each phlebotomist.
