# Supplementary figures and images for: Hemoglobin concentration and anemia diagnosis in venous and capillary blood: biological basis and policy implications
Source: Ann N Y Acad Sci. 2019 Jun 23;1450(1):172–89. doi: 10.1111/nyas.14139 (PMC7496102; doi:10.1111/nyas.14139)

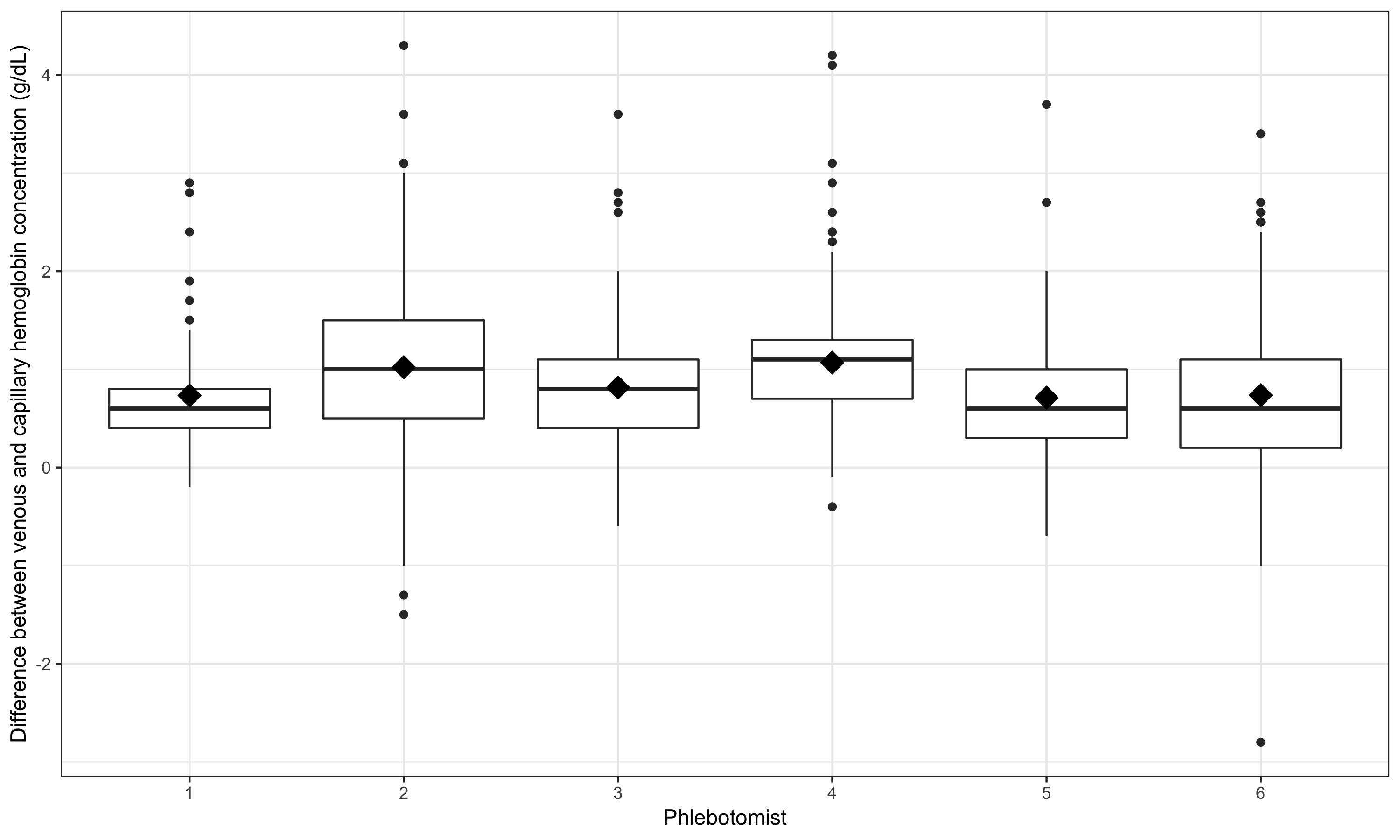

Supplement: Supplementary file 1 — Figure S1. Box plot of mean differences between venous and capillary samples by a phlebotomist. Horizontal box lines represent median and interquartile ranges. Diamonds represent means hemoglobin concentrations for each phlebotomist. [file NYAS-1450-172-s001.tiff]
